# Porous polyetheretherketone microcarriers fabricated via hydroxylation together with cell-derived mineralized extracellular matrix coatings promote cell expansion and bone regeneration

**DOI:** 10.1093/rb/rbab013

**Published:** 2021-03-19

**Authors:** Shuo Sun, Zixue Jiao, Yu Wang, Zhenxu Wu, Haowei Wang, Qingming Ji, Yi Liu, Zongliang Wang, Peibiao Zhang

**Affiliations:** 1 Department of Spine Surgery, The First Hospital of Jilin University, 1 Xinmin Street, Changchun 130021, China; 2 Key Laboratory of Polymer Ecomaterials, Changchun Institute of Applied Chemistry, Chinese Academy of Sciences, 5625 Renmin Street, Changchun 130022, China

**Keywords:** polyetheretherketone, porous microcarriers, mineralized extracellular matrix, cell expansion, bone regeneration

## Abstract

Porous microcarriers have aroused increasing attention recently by facilitating oxygen and nutrient transfer, supporting cell attachment and growth with sufficient cell seeding density. In this study, porous polyetheretherketone (PEEK) microcarriers coated with mineralized extracellular matrix (mECM), known for their chemical, mechanical and biological superiority, were developed for orthopedic applications. Porous PEEK microcarriers were derived from smooth microcarriers using a simple wet-chemistry strategy involving the reduction of carbonyl groups. This treatment simultaneously modified surface topology and chemical composition. Furthermore, the microstructure, protein absorption, cytotoxicity and bioactivity of the obtained porous microcarriers were investigated. The deposition of mECM through repeated recellularization and decellularization on the surface of porous MCs further promoted cell proliferation and osteogenic activity. Additionally, the mECM coated porous microcarriers exhibited excellent bone regeneration in a rat calvarial defect repair model *in vivo*, suggesting huge potential applications in bone tissue engineering.

## Introduction

The bone defects, originated from trauma or disease, are a great challenge in the field of bone tissue engineering [1, 2]. An ideal bone substitute material should support cellular growth and the formation of extracellular matrix (ECM) [3]. Presently, a variety of well-developed scaffolds based on metal, alloy, natural or synthetic polymers are widely applied [4–7]. In the last two decades, although great progress has been made in bone reconstruction and regeneration, the traditional bulk scaffolds have a limitation of the cell loading area and therefore remain futile in irregular and complex defects [8–11].

Recently, microcarriers (MCs) are receiving much attention for their wide application in cell therapy and tissue engineering [12–14]. Compared to the traditional planar cell culture system, MCs possess desirable advantages of maintaining the differential cell phenotype, direct injection and better cell delivery to facilitate the repair of irregular surgical defects [15, 16]. Notably, compared to the solid spherical MCs with smooth surfaces, the porous MCs with interconnected pores offer a larger surface area enabling nutrient and oxygen transfer, cell attachment and cell growth. These are among the vital requirements for sufficient cell seeding density [14, 17, 18].

MCs can be prepared using both natural polymers and synthetic polyesters. Previously, based on the synthetic polyetheretherketone (PEEK), we developed smooth surface MCs that showed excellent cytocompatibility and chemical resistance [19]. However, due to the natural inertness of PEEK, these MCs had biological limitations in bone tissue engineering [20]. PEEK has been used more recently in calvarial reconstruction [21–24]. Its advantage lies in minimal imaging artifact, being nonmagnetic, lightweight and an inert nonconductor [25]. Unfortunately, the bioinertness nature and inferior osteoinduction property of PEEK still hamper its clinical adoption [26, 27]. 3D printed PEEK has been used as patient-specific implant, but it is also difficult to repair irregularly shaped defects with a prefabricated form, especially for the application in emergency operation [28]. Therefore, PEEK material fabricated in the form of MCs may fill any defect cite, regardless of its geometry and integrates with host tissue after appropriate modification, finally simplifying the design and surgical procedure [18].

Tailored MCs with appropriate physicochemical properties are suggested to influence some of the critical events in tissue repair such as immune response, angiogenesis, grafting and differentiation [29–31]. For example, the surface chemistry altered by functional groups and nano-/micro-patterned surfaces greatly influences cell adhesion, migration and differentiation [32, 33]. Since PEEK is chemically inert and has a relatively hydrophobic surface, it is not optimal for protein absorption [22]. Importantly, hydroxylation of PEEK can moderately increase its surface hydrophilicity influencing cell adhesion through protein absorption [20, 34]. Also, the porous surface structure, a special kind of topological morphology, can contribute to cell adhesion and differentiation [14, 31]. Furthermore, to bring cell recognition cites onto the hydroxylated porous MCs, mineralized extracellular matrix (mECM) could provide a physiological microenvironment conductive to cell proliferation, intracellular communication, collagen secretion and osteogenic differentiation [35–37]. Therefore, by combining mECM and porous MCs, bioactive PEEK MCs can offer both osteoconductivity and osteoinductivity in bone regeneration.

**Figure 1.**
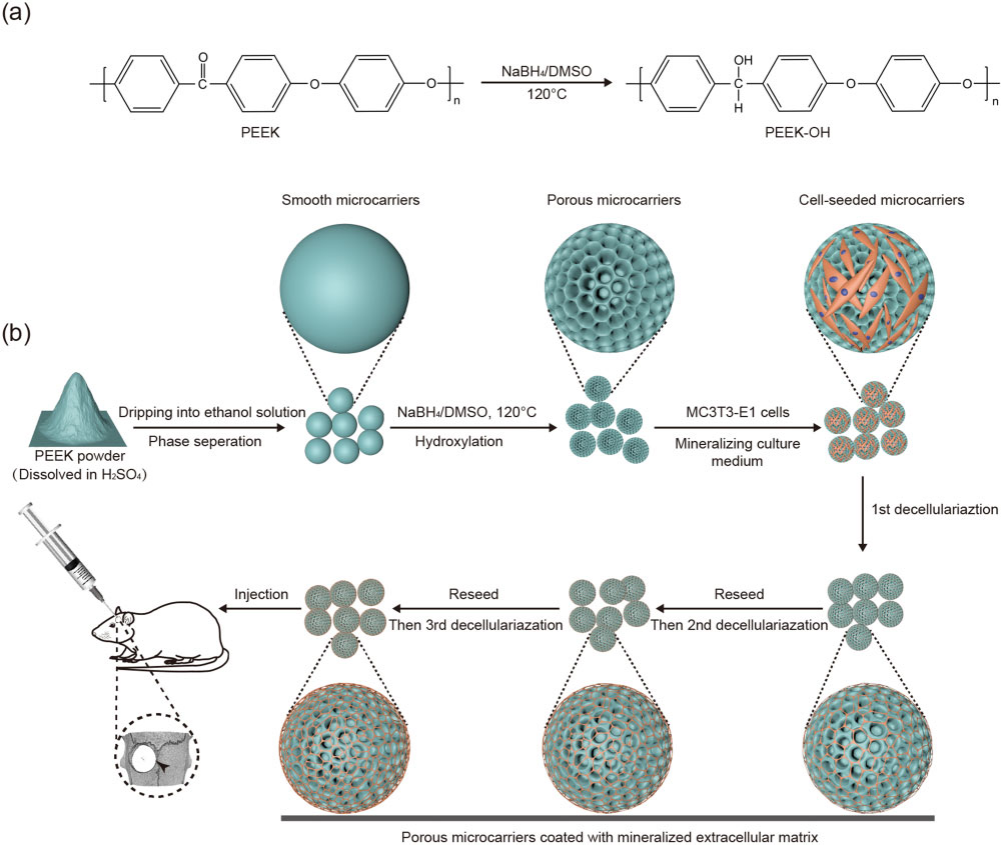
Schematic illustration for the fabrication of porous and mineralized ECM coated PEEK microcarriers. (**a**) The hydroxylation of microcarriers was achieved through the reduction reaction of the carbonyl groups. (**b**) The smooth microcarriers were first prepared with liquid–liquid phase separation method. Porous PEEK microcarriers were then derived from smooth microcarriers by hydroxylation. Next, MC3T3-E1 cells were cultured on the porous microcarriers with mineralizing culture medium, containing regular medium supplemented with Ca^2+^ and PO4^3−^. The cell-seeded porous microcarriers were then treated with three rounds of decellularization to get denser mineralized extracellular matrix mesh. Finally, mineralized ECM coated porous microcarriers were injected into the calvarial defects of the rats to evaluate bone regeneration *in vivo*

Here we prepared porous PEEK MCs from smooth MCs by reduction of carbonyl groups (Fig. 1) and evaluated them for microstructure, protein absorption, cytotoxicity and bioactivity. Besides, by repeated decellularization and recellularization, mECM was successfully deposited on the surface of porous MCs, which further promoted cell adhesion, proliferation and osteogenic differentiation *in vitro*. Our *in vivo* results suggested that these modified MCs delivered excellent bone regeneration in a rat model of critical-sized calvarial defects.

## Materials and methods

### Materials and reagents

PEEK powder was acquired from Victrex (England). Sodium borohydride (NaBH_4_, 99%) was purchased from Beijing Chemical Works (China). Dimethyl sulfoxide (DMSO) was purified with distillation from calcium hydride (Aladdin) before use. All other chemicals and solvents were of analytical grade or higher and used as received.

### Preparation of smooth and porous PEEK MCs

Preparation of smooth MCs was performed as described previously [19] and the details of the preparation process are available in the Supplementary data. Porous PEEK MCs were first prepared utilizing the simple wet-chemistry method with NaBH_4_ dissolved in DMSO at 120°C. The resulting PEEK MCs with porous surface microstructure were achieved through the reduction reaction of the carbonyl groups according to the literature [38]. Briefly, 60 ml of freshly distilled DMSO and 120 mg of NaBH_4_ were added to a dried reactor and heated to 120°C. After total dissolution, 0.1 g dried smooth PEEK MCs were immersed in the gently stirred reaction mixture and heated at 120°C for 3 h under nitrogen. After removing from the reaction mixture, the PEEK MCs were successively rinsed with methanol (15 min), distilled water (10 min), 0.5 M HCl (10 min), distilled water (10 min) and ethanol (10 min). The porous MCs were then thoroughly dried at room temperature under vacuum and stored under nitrogen in the dark.

### Characterization of MCs

The surface micromorphology of different PEEK MCs was examined by scanning electron microscope (SEM, XL30 FEG, Philips). All the PEEK MCs were sputter-coated with gold in advance. SEM coupled with an EDX detection system (detector X-Max^N^, Oxford Instruments, UK) was further used for mineral evaluation, atom inspection, and elemental identification of the deposited mECM.

Mercury intrusion porosimetry was carried out with an Auto Pore IV 9500 (Micromeritics, USA) to investigate the pore structure and property.

Fourier Transform Infrared Spectrometer (FTIR, Bio-Rad Win-IR spectrometer, UK) was used to detect chemical groups. The FTIR spectra were carried out in the wavelength range between 600 and 4000 cm^−1^ with a resolution of 2 cm^−1^.

For protein absorption capability, samples of smooth and porous MCs (0.01 g) were pre-equilibrated with 1 ml phosphate buffer solution (PBS, pH range 4.7–8.4) in tubes overnight at 37°C. Bovine serum albumin (BSA, Solarbio, 1 mL, 2 mg/mL) was added to the tubes after discarding PBS. The tubes (in triplicate) were shaken at 150 rpm, 37°C until equilibrium. The amounts of BSA concentration loaded in the MCs were measured by determining the concentration reduction in the supernatant, which was analyzed by BCA protein assay kit (Thermo Fisher, USA, wavelength = 562 nm).

The static water contact angles of the smooth and porous MCs were measured by the sessile drop method on a contact angle system (Kruss, DSA100, Germany). Each different sample was measure at three separate points.

### *In vitro* cytocompatibility evaluation

#### Cell culture

*In vitro* cell study was carried out using the MC3T3-E1 cell, which was obtained from Institute of Biochemistry and Cell Biology, Shanghai Institutes for Biological Sciences, Chinese Academy of Sciences. Cells were maintained in Dulbecco’s modified Eagle’s medium (DMEM, Gibco) supplemented with 10% FBS (Gibco), 5 ml penicillin–streptomycin solution (100×, Solarbio) at 37°C in 5% CO_2_. The medium was refreshed every 2 days.

#### Cytotoxicity test

The cytotoxicity test *in vitro* was performed based on the International Standard for the biological evaluation of biomedical devices (ISO 10993-5), the experimental details are available in the Supplementary data.

#### Cell adhesion

To investigate the effect of surface modification of PEEK MCs on cell adhesion, MC3T3-E1 cells at a density of 2 × 10^4^ were inoculated with MCs in a 48-well tissue culture plate and cultured for 1, 3 and 7 days, respectively (*n* = 3). Double staining methods with Calcein AM (Sigma) for live cells (green) and 4,6-diamidino-2-phenylindole (DAPI, Invitrogen) for cell nucleus (blue) were performed. The stained cells were observed under a fluorescent microscope (TE2000U, Nikon, Japan).

#### Cellular morphology and ECM secretion

Cell morphology and ECM secretion of MC3T3-E1 cells grown on smooth and porous MCs were visualized by SEM. Briefly, cells were seeded on MCs in the 48-well tissue culture plate at a density of 2 × 10^4^ cells per well and cultured for 1, 3 and 7 days. At each prescribed time point, the culture medium was discarded, and the MCs were rinsed with PBS and fixed in 4% paraformaldehyde (PFA) solution for 20 min. Afterwards, the MCs loaded with cells were dehydrated in graded ethanol series. Finally, the critically dried samples were sputter-coated with gold and observed by SEM.

#### Cell proliferation

Cell proliferation Reagent Kit (CCK-8, 7 Sea Biotech, Shanghai, China) was used to assess cell proliferation. The details of the cell proliferation assay are available in the Supplementary data.

#### mECM formation

Dry porous MCs were first hydrated in PBS for at least 3 h. The supernatant was decanted, and the porous MCs were washed twice with fresh PBS and then sterilized by autoclaving at 121°C for 20 min. The autoclaved porous MCs were then equilibrated in a cell culture medium at 37°C overnight before inoculation. Finally, the porous MCs were suspended in regular DMEM and evenly mixed with the MC3T3-E1 cells at a density of 5 × 10^4^ in the 48-well plate per well. After 2 days of culture, the medium was replaced with a mineralizing medium containing a regular DMEM supplemented with 9 × 10^−3^ M CaCl_2_, 4.2 × 10^−3^ M K_2_HPO_4_ and 0.2 mg/mL polyaspartic acid. The mineralizing medium was changed 24 h later and then refreshed every 2 days. After incubation at 37°C in 5% CO_2_ for 5 days, the cell-seeded porous MCs were collected for use in the following experiments.

#### Decellularization and mECM morphology

The decellularization method was performed according to the literature [39]. The collected cell-seeded porous MCs were rinsed with PBS and decellularized by treating with 20 mM NH_4_OH/0.25% Triton X-100 at room temperature for 10 min. The resulting MCs were rinsed twice with PBS. For SEM imaging, the samples were fixed with 4% PFA, washed with PBS, and then dehydrated through a graded series of ethanol. Finally, after air drying, the morphology and element analysis of the samples were evaluated by the SEM and coupled EDX detection system. The residual samples were air-dried overnight and stored at −20°C for subsequent recellularization.

#### Recellularization and cell adhesion, proliferation evaluation

To acquire denser and widespread mECM coatings, part of the porous MCs coated with mECM after first decellularization was reseeded and decellularized as aforementioned for a second and third time. Finally, the mECM-coated porous MCs after different times of decellularization were again used for cell adhesion and proliferation evaluation.

### Osteogenic differentiation evaluation *in vitro*

Taking the time and cost factors into consideration, only the porous MCs coated with mECM after first decellularization were used for osteogenic differentiation evaluation in this section unless otherwise specified. Experimental details of the following procedures are available in the Supplementary data.

#### ALP staining and activity

The MC3T3-E1 cells at a density of 5 × 10^4^ cells per well (*n* = 3) were evenly mixed with the samples and seeded in the 48-well plate using DMEM cell culture medium. After incubation for 7 days, alkaline phosphatase (ALP) staining was evaluated by using kits purchased from Beyotime (Shanghai, China).

#### Alizarin red staining calcium deposition assay

The MC3T3-E1 cells were seeded on MCs in a 48-well culture plate at a density of 5 × 10^4^ cells per well (*n* = 3) using DMEM cell culture medium. The medium was changed every 2 days. The alizarin red staining and calcium deposition assay were performed after 14 days of incubation.

#### Quantitative real-time polymerase chain reaction (qRT-PCR)

To analyze the gene expression profile, the MC3T3-E1 cells were seeded onto the smooth, porous and mECM coated porous MCs at a density of 10 × 10^4^ cells in a 6-well plate per well. The qRT-PCR assay was performed after 7 days of incubation.

### *In vivo* animal study

#### Animal models

The animal study was approved by the Laboratory Animal Welfare & Ethics Committee, College of Basic Medical Sciences, Jilin University (Changchun, China). Critical-sized calvarial bone defects 5 mm in diameter were created in Sprague–Dawley rats (180–200 g, 6- to 8-weeks old) to evaluate the capability of different PEEK MCs for bone substitution and regeneration. Twenty-four rats were randomly assigned into four groups of six animals: untreated (control), implanted with smooth MCs, implanted with porous MCs and implanted with mECM coated MCs (only after first decellularization), respectively. The rats were euthanatized at 4 and 8 weeks after implantation (12 rats at each time point). The calvarial bones were obtained and fixed with 4% PFA for further analysis.

#### Microcomputed tomographic analysis

All the fixed samples were scanned in a micro-CT system (SkyScan 1172; Bruker, Belgium), using 80 kV and 100 μA with a 0.5 mm aluminum filter. The NRecon and CTvox softwares (Bruker) were used for 3D reconstruction, and the CTAn software (Bruker) was applied to calculate the ratio of bone volume to tissue volume (BV/TV). The measurement was made in triplicate by an experienced researcher blinded to the group assignment.

### Statistical analysis

Statistical analysis was conducted using analysis of variance (ANOVA, one-way, GraphPad Prism 7, USA). Triplicate samples were analyzed in each experiment. All the experimental data were expressed as mean ± standard deviations (SDs). Statistically significant differences were set at *P* ≤* *0.05.

## Results and discussion

### Preparation and characterization of porous MCs

**Figure 2.**
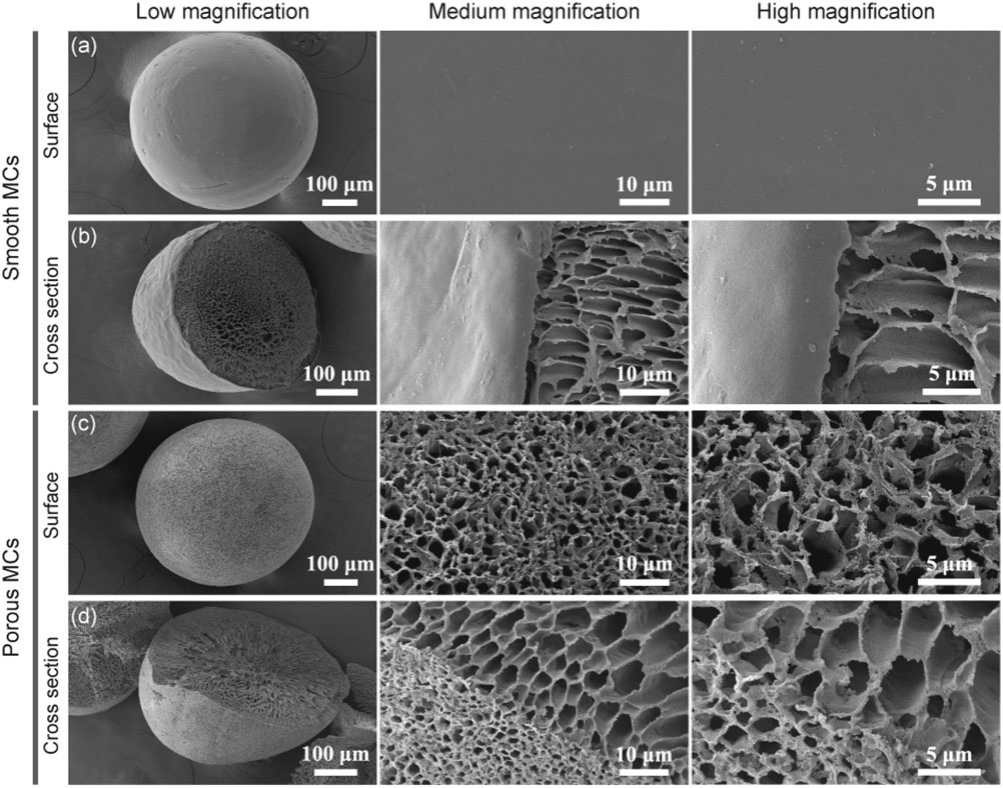
SEM micrographs of smooth (**a and b**) and porous (**c and d**) microcarriers. Both of the microcarriers were first fabricated with liquid–liquid phase separation method by dripping PEEK/H_2_SO_4_ into 30% ethanol solution at 0°C. The porous microcarriers were derived from smooth microcarriers by hydroxylation treatment

The surface and cross-section microstructure of the smooth and porous MCs are shown in Fig. 2. The smooth MCs already had a honeycomb-like pore structure (Fig. 2b). The smooth MCs were fabricated by the liquid–liquid phase separation technique, involving the precipitation of PEEK in a non-solvent. The PEEK solution was dripped into distilled water (DW)/ethanol (Eth) co-medium. The rapid exchange of the solvent sulfuric acid and DW/Eth co-medium triggered a rapid liquid–liquid phase separation to form a skin layer [40]. Upon complete exchange between the solvent and the non-solvent, the generated PEEK MCs exhibited a smooth outside surface and a porous inside structure.

Chemical modifications of PEEK have become an interesting tool to develop biomaterials with specific physicochemical properties for novel applications [41]. For instance, its hydroxylated derivatives (PEEK-OHs) exhibit increased reactivity [42] due to high amounts of hydroxyl functions and are widely used as a key intermediate for constructing designed surfaces [43]. PEEK-OH also promotes cell adhesion and proliferation [20, 34]. In this study, by using the wet-chemistry method, the reduction of the carbonyl groups resulted in a uniform micropore structure on the surface of MCs. Since the reduction from ketone to hydroxyl group rendered loss in the thermal stability [44], the surface layer of smooth MCs was dissolved in hot DMSO for a longer reaction time and a higher degree of hydroxylation. Finally, the inner pore structure gets exposed. This destabilization attributes to a decrease in the resonance effect due to the presence of sp^3^ carbons and the altered spatial organization of the molecules [41].

As measured by mercury porosimeter, the median and average pore diameters were 3171.01 nm and 569.72 nm, respectively (Table 1). The SEM images revealed that the pores in the porous MCs were larger in the center while turned smaller closer to the outer surface, suggesting a hierarchical micro/nanopore structure (Fig. 2c and d).

**Table 1.** Physical properties of the porous MCs measured by mercury porosimeter

Properties	Porous MCs
Pore type	Nano and micropore
Median pore diameter, nm	3171.01
Average pore diameter, nm	569.72
Porosity, %	90.3183
Bulk density, g/ml	0.0935
Skeletal density, g/ml	0.9659
Surface area, m^2^/g	67.807

**Figure 3.**
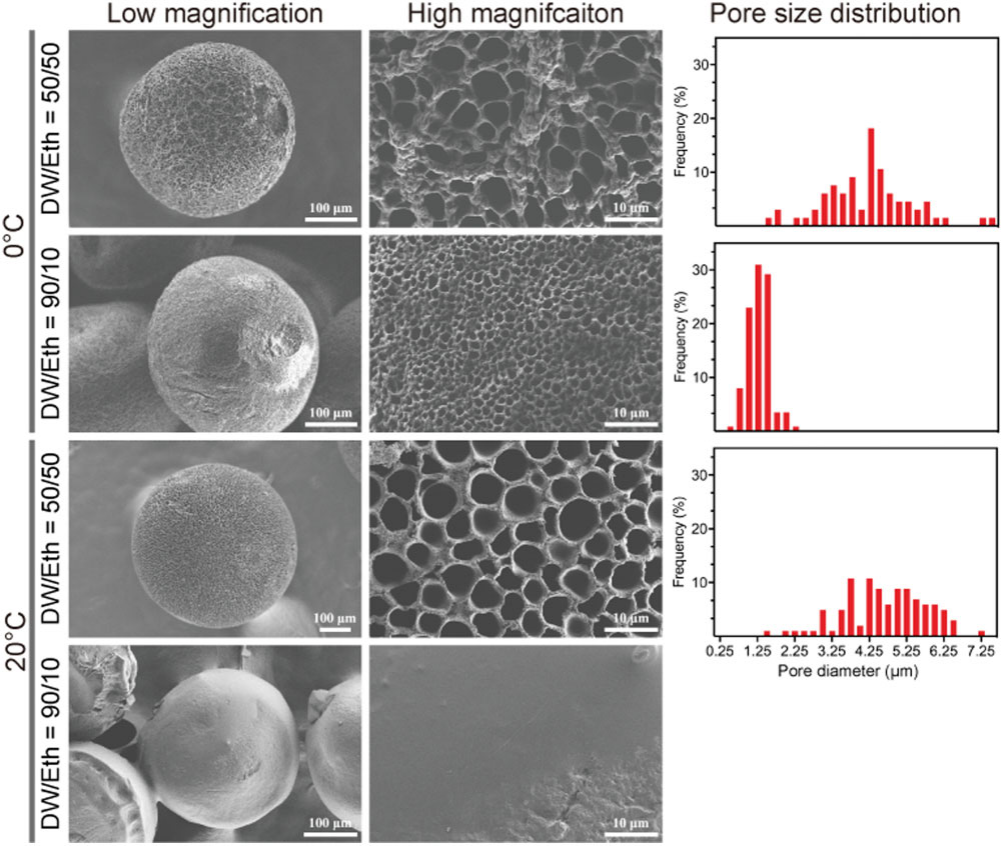
Surficial microstructures and pore size distribution of the porous microcarriers produced by various processing conditions

The size of the micropores on the surface of porous MCs can be regulated by adjusting various processing parameters such as exchange solution composition and temperature. The microstructure and pore size distribution of MCs prepared in different conditions are presented in Fig. 3. We found that in comparison to temperature, the co-medium ratio of DW/Eth was more critical in determining the pore size. For instance, at the same temperature, the micropore size turned smaller (from 4.16 ± 1.17 μm to 1.26 ± 0.30 μm at 0°C) with an increase in the DW/Eth ratio. Notably, at 20°C, a co-medium DW/Eth ratio of 90/10 failed to generate any pores. As per the nucleation theory [40, 45], a higher ratio of DW/Eth should facilitate sulfuric acid nucleation during solidification, which ultimately downsizes the micropores. An altered constitution and temperature of the co-medium tend to collapse the inner structure of the resulting MCs, which cannot sustain the hydrothermal treatment or hydroxylation process. Here, after successful optimization of the processing parameters, DW/Eth co-medium having a ratio of 70/30 at 0°C was found to be the best conditions for MCs preparation.

**Figure 4.**
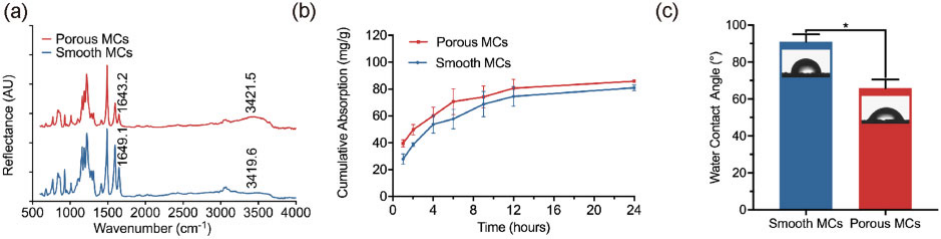
FTIR spectra characterization (**a**), the kinetics of protein absorption (**b**) and static water contact angle (**c**) of smooth and porous microcarriers. *P ≤ 0.05, *n* = 3 for each group. The protein absorption of microcarriers was tested in 2 mg/ml BSA solution (pH = 7.35, 37°C).

Next, the surface chemical modification of the porous MCs was verified by FTIR spectra (Fig. 4a). We noticed that compared to the smooth MCs, after hydroxylation, the band around 1650 cm^−1^ related to the stretching vibration of the carbonyl group of the benzophenone segment was significantly reduced in the porous MCs. Moreover, a new band at around 3420 cm^−1^ appeared which refers to the stretching vibration of the alcoholic hydroxyl group. These results confirmed that the hydroxyl groups, derived from the reduction of the carbonyl groups, were successfully formed in porous MCs.

Model protein BSA was used to determine the protein absorption onto the smooth and porous MCs. The corresponding time-absorption curves are shown in Fig. 4b. After soaking in BSA solution for 12 h, the absorption curves reached the plateau phase. The equilibrium capacity of smooth and porous MCs was 74.53 ± 7.27 mg/g and 80.67 ± 6.47 mg/g, respectively, suggesting an improved protein loading capacity of porous MCs. A higher surface area and modified hydrophilicity led to an increase in protein absorption. Besides, the hierarchical pore system improved protein absorption capacity due to increased protein holding in nanoscale pores [46].

The static water contact angle measurement was used to compare the hydrophilicity of smooth and porous MCs (Fig. 4c). The water contact angle was 91.08 ± 3.88° for the smooth MCs, whereas it decreased to 65.88 ± 4.55° after hydroxylation. In order to protect the surface topography of the smooth and porous MCs, we used the double-sided tape to stabilize a single densely stacked layer of MCs instead of tableting for the water contact angle measurement. The results of the water contact angle measurement also verified that the surface of smooth MCs has been modified successfully. The hydroxylation process could obviously improve the surface hydrophilicity of PEEK MCs and may further enhance biological activity.

### *In vitro* cytocompatibility evaluation

#### Cytotoxicity

**Figure 5.**
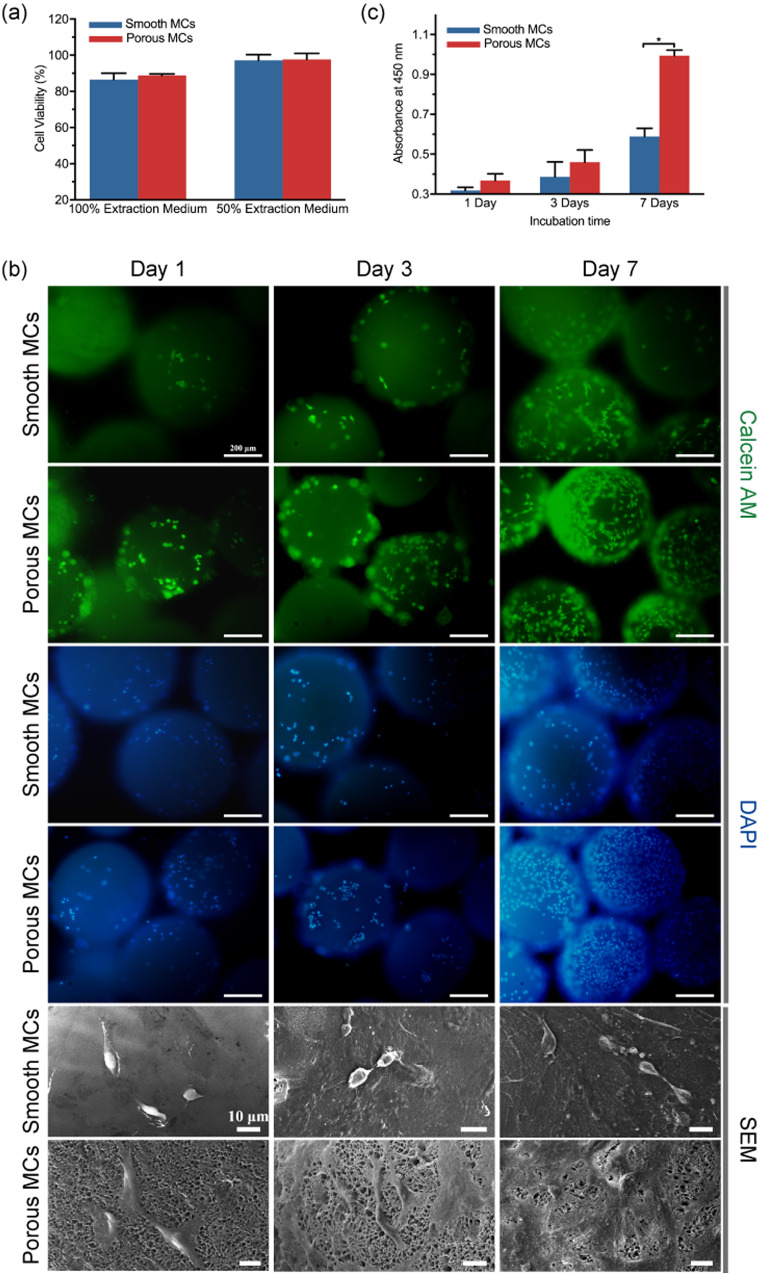
*In vitro* cytocompatibility evaluation of smooth and porous microcarriers. (**a**) Cell viability was evaluated by culturing MC3T3-E1 cells in smooth and porous microcarriers extracts for 24 h through CCK-8 assay kit. (**b**) Fluorescent and SEM micrographs of MC3T3-E1 cells on smooth and porous microcarriers at 1, 3 and 7 days post seeding. The cells were stained with Calcein AM (cytoplasm, green) and DAPI (nucleus, blue). The scale bar lengths are 200 μm and 10 μm, respectively. (**c**) Proliferation of MC3T3-E1 cells on smooth and porous microcarriers at 1, 3 and 7 days post seeding were evaluated by CCK-8 assay kit (*P ≤ 0.05, *n* = 3 for each group)

Before the cytological evaluation, the cytotoxicity of the extracts from smooth and porous MCs was assessed (Fig. 5a). For smooth and porous MCs in 100% extracts, the cell viability was 88.53 ± 3.52% and 88.75 ± 0.94%, respectively. For the extract, diluted by 1-fold, the cell viability changed to 97.19 ± 3.18% and 97.70 ± 3.31%, respectively. We found no significant difference between 100 and 50% extracts. Though both of the MCs were originally prepared from PEEK powder dissolved in concentrated sulfuric acid, the subsequent hydrothermal treatment significantly decreased the sulfur content [27, 47]. The NaBH_4_ and DMSO used in the preparation of porous MCs were also removed by thoroughly washing. The indirect cytotoxicity assay indicates that both the smooth and porous MCs are safe for *in vivo* applications.

#### Cell adhesion

The MC3T3-E1 cells adhered to the samples after incubation for 1, 3 and 7 days are shown in Fig. 5b. A relatively hydrophobic surface and the low surface energy is an intrinsic problem of PEEK material [48], which limits the cellular adhesion by affecting the cell adhesion related proteins such as fibronectin and vitronectin [49]. Thus, increasing surface energy has a positive impact on initial reactions between cells and the PEEK material [50, 51]. Among the various ways to change the surface energy, wet-chemical treatment involving direct surface modification is a widely used, reproducible, uniform and stable method [48]. In this study, using the one-step hydroxylation treatment simultaneously changed the surface chemistry and structure of the smooth MCs. The hydroxylated PEEK, being less hydrophobic than pure PEEK [20, 52], is beneficial for improving cell adhesion by reorienting fibronectin [53]. Both surface hydrophilicity and topography greatly influence cell–material interactions [54–56]. As shown by the DAPI nuclear staining (Fig. 5b), more cells were attached to porous MCs than smooth MCs at all the assessed time points. Notably, this result is consistent with the proliferation assay (Fig. 5c). Concerning cell morphology, compared to the smooth MCs, the cells on the surface of porous MCs were well spread and exhibited improved density as revealed by Calcein-AM staining.

#### Cellular morphology and ECM secretion

Cellular morphology of MC3T3-E1 cells, cultured for 1, 3 and 7 days was visualized by SEM (Fig. 5b). After incubation for 1 day, the cell morphology exhibited significant differences between the smooth and porous MCs. MC3T3-E1 cells adhered to the smooth MCs were of a spindle or spheroidal shape and lack of lamellipodia showing the characteristics of the original pre-osteoblast, whereas cells adhered to the porous MCs were typically flat, highly elongated with protruding filopodia. This demonstrates the bio-inertness of the smooth pure PEEK surface.

After 3 days of incubation, MC3T3-E1 cells grown on the smooth MCs maintained spheroidal shape with sparse ECM coated on the surface. However, cells grown on the porous MCs were well spread with higher elongation ratios and filopodia protrusions. Also, the ECM coating was denser than the cells of smooth MCs, suggesting healthy cell growth and exuberant activity.

After 7 days of incubation, the differences in the cell morphology and ECM secretion became further evident. The MC3T3-E1 cells grown on the smooth MCs transformed from spheroidal shape to round flat shape while some of the cells still showed fabiform morphology. In contrast, cells grown on the porous MCs featured numerous filopodial and lamellipodial and were well buried in the dense and widespread ECM.

#### Cell proliferation

Next, we evaluated the proliferation of the MC3T3-E1 cells by CCK-8 assay (Fig. 5c). We noticed the absorbance increased with longer culture time indicating pre-osteoblasts proliferation. However, cell proliferation was better for the porous MCs group at all assessed times, especially on day 7 (*P* < 0.05). This suggests that improved cell proliferation was evident for porous MCs.

Surface morphology and chemical composition, the key features of a biomaterial, directly influence the initial interactions between cells and implant surface [57]. Our *in vitro* study revealed that cell adhesion, spreading, ECM secretion and proliferation were significantly different between smooth and porous MCs. The cellular response was enhanced for porous MCs as the porous structure and moderate hydrophilic surface ensured initial cell adhesion and subsequent spreading, proliferation and ECM secretion, which were promising in generating a larger volume of new bone, and stronger bone/implant bonds *in vivo*. Lee *et al*. [58] showed that the pore diameter, number of pores per mm^2^, and average pore to pore distance can affect cell adhesion and proliferation, although different cell types may react differently depending on the surface topography.

#### mECM formation and recellularization

**Figure 6.**
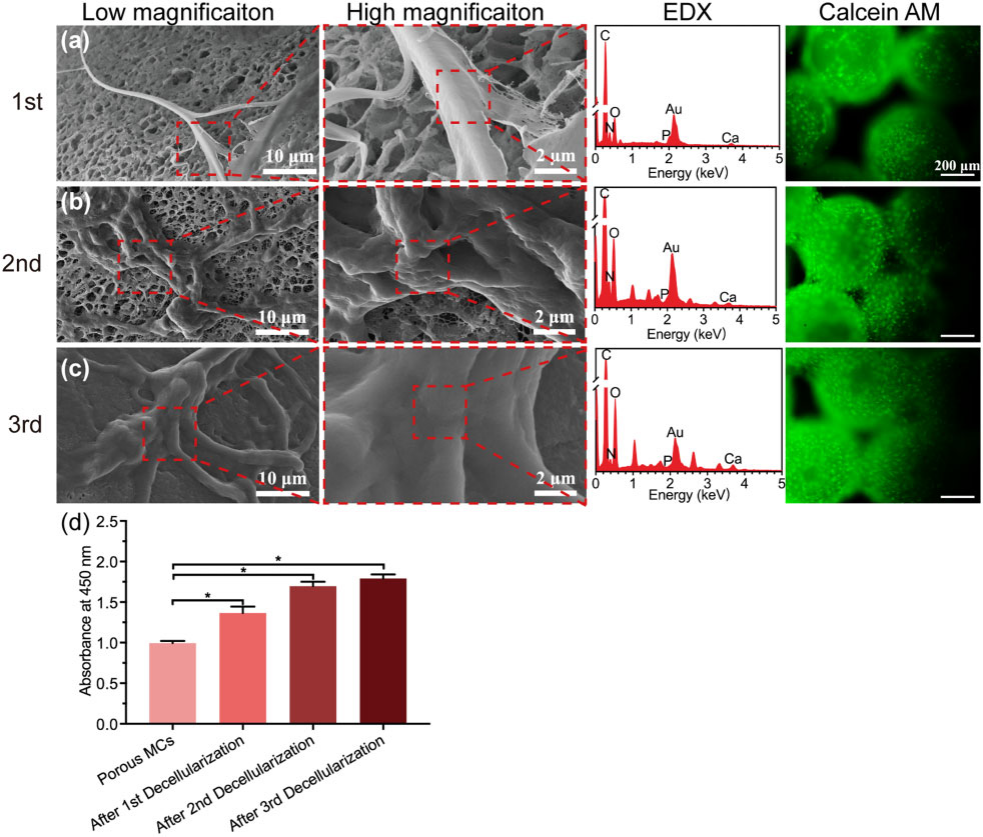
Characterization and recellularization evaluation of the mECM coated porous microcarriers. The surface microscopic morphology, elemental analysis and cell adhesion of porous microcarriers after first (**a**), second (**b**) and third (**c**) decellularization were evaluated. For cell adhesion assay, the MC3T3-E1 cells were stained with Calcein AM (cytoplasm, green, all scale bar lengths are 200 μm). (**d**) After 7 days of incubation, the proliferation of MC3T3-E1 cells on porous microcarriers of different decellularization times was evaluated by CCK-8 assay kit (*P ≤ 0.05, *n* = 3 for each group)

Even though the porous MCs showed enhanced cell adhesion, better cell spreading and proliferation, lack of cell recognition sites may limit its applications. Since exuberant ECM secretion is believed to improve cell–cell recognition [59–61], cell-derived ECM coating on the surface of porous MCs through repeated decellularization strategy was used to further improve cytocompatibility. To aid bone regeneration, we fabricated mECM to mimic natural bone structure and microenvironment. For the different times of decellularization, the microstructure, elemental analysis and the cell adhesion properties of ECM are shown in Fig. 6a–c. We noticed that mECM was better widespread and denser with increasing decellularization times (also in Supplementary Fig. S1). The elemental analysis confirmed the ECM components (nitrogen) and the presence of mineral phase with calcium and phosphate. Thermogravimetric analysis (TGA, TA Instruments TGA500, USA) was also used to quantitatively measure the ECM amount and the results are shown in Supplementary Fig. S2. Compared with conventional crystallization, the polymer-induced liquid-precursor mineralization process in this study could result in intrafibrillar mineralization [62, 63] mimicking the natural bone structure and environment. Also, the ECM formation and mineralization could be completed in a single round of culture where the introduction of calcium not only contributed to mineralization but also ECM formation [64–66]. Thus, the procedure is very time-saving and cost-efficient.

Based on the past literature, exposures to nonionic detergents or chelating agents are the two main methods for decellularization [67]. To maintain the mineralization components within the mECM, instead of using the chelating agents, the chemical process using alkaline reagent and detergent were selected in this study. This method could also efficiently remove the cellular components that may cause adverse immune reactions [68].

Our PEEK MCs can endure high temperature and pressure and therefore can be subjected to autoclaving for repeated seeding with MC3T3-E1 cells. Besides, the mECM further improved the surface hydrophilicity, which in turn promotes cell–cell communication [37, 69], cell attachment and proliferation. We found that an increase in decellularization times enhanced cell adhesion and the cells were uniformly distributed on the porous MCs. Especially, after third decellularization, the surface of porous MCs was almost completely covered with cells (Fig. 6c). After 7 days of cell incubation, compared to the original ones, decellularized MCs indicated better cell proliferation in mECM coated porous MCs (Fig. 6d).

### Osteogenic differentiation evaluation

#### ALP staining and activity assay

**Figure 7.**
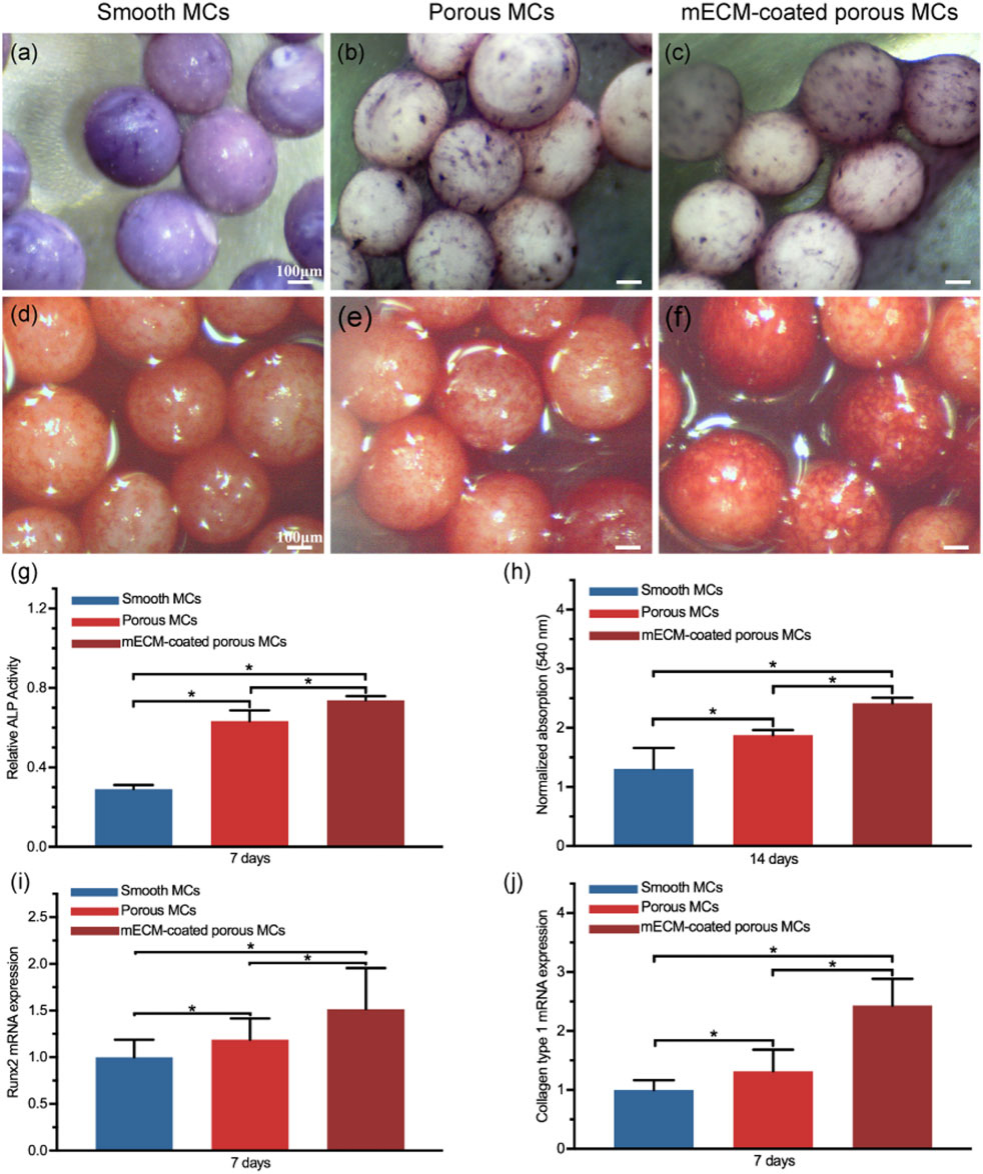
Osteogenic differentiation evaluation. ALP staining (**a–c**) and activity (g) after incubation for 7 days, alizarin red staining (**d–f**) and amount of calcium deposition (h) after incubation of MC3T3-E1 cell for 14 days on different samples. qRT-PCR analysis of Runx2 (i) and Col-I (**j**) of MC3T3-E1 cells grown on different microcarriers after incubation for 7 days (*, *P* ≤ 0.05, *n* = 3 for each group, all scale bar lengths are 100 μm)

After 7 days of incubation, ALP staining and activity analysis were performed to evaluate the effects of the MCs on osteogenic differentiation. MC3T3-E1 cells were employed for *in vitro* tests in this study because they can differentiate into osseous tissue as pre-osteoblast cells. The cells adhered to the MCs were visualized by ALP staining under a stereomicroscope. We found that compared to the porous MCs (Fig. 7b), a deeper violet color was observed on the mECM coated MCs (Fig. 7c), which was consistent with an increased ALP activity (Fig. 7g). A significantly lower ALP activity and less normal cellular morphology of the smooth MCs were detected when compared within the groups. However, the smooth MCs showed a deeper violet color in staining (Fig. 7a), suggesting nonspecific absorption of ALP stain.

#### Alizarin red staining and calcium deposition assay

After 14 days of incubation, only nonconsecutive and lightly stained nodules could be observed on smooth MCs (Fig. 7d), which indicated that smooth MCs barely possess favorable osteoinduction ability. In the porous MCs group, the presence of denser and deeper red nodules suggested that surface morphology modification effectively promoted the bone induction (Fig. 7e). Meanwhile, mECM coated MCs group exhibited improved calcium deposition (Fig. 7f). Furthermore, the quantification of mineral deposits was performed to verify the staining results. We found that the total calcium content was higher in the mECM coated porous MCs (Fig. 7h). Also, the calcium contents of porous MCs groups were significantly higher than the smooth MCs. The above results suggest that the mECM and porous morphology enhanced bone induction and calcium deposition synergistically.

#### qRT-PCR analysis

To further strengthen the above-said results, we evaluated the expression levels of runt-related transcription factor 2 (Runx2) and collagen type I (Col-I) using qRT-PCR after 7 days of incubation (Fig. 7i–j). The results showed that the mECM coated porous MCs substantially increased the mRNA expression levels of all markers compared to the smooth and porous MCs.

3D porous structures and chemical composition can be used to determine the osteoinductivity of materials [54–56]. Generally, an increase in surface roughness promotes osteoblasts differentiation compared to a smoother surface [58]. Osteoinductivity is a general feature of porous PEEK films and rods, while the smooth PEEK cannot induce bone formation [48]. The ALP activity, calcium deposition and the expression of both bone-related genes Runx2 and Col-I were higher in the porous MCs compared to the smooth MCs. The porous structure of the MCs promoted osteoinduction. It has been shown that the surface micropores <10 μm in the implanted scaffolds facilitated both the penetration of body fluids, cell adhesion and osteogenic differentiation due to an increase in surface roughness [70–72]. The internal nanoscale pores confine the flow of body fluid to create a locally high concentration of Ca^2+^ and ${\text{PO}}_{4}^{3-}$, which is favorable for osteoinduction [73]. Also, the porous structure facilitates the absorption of low molecular weight proteins [46]. All of these could be the potential reason for better osteoinductivity in porous MCs.

Compared with the porous MCs, the mECM coatings on the porous surface further enhanced osteogenesis, as evident from greater ALP activity, more calcium deposition, and the upregulation of related genes. ECM microenvironment regulates many cellular functions. *In vitro* study shows that cell-derived ECM is a promising material for tissue engineering. Also, decellularization removes antigenic epitopes that may cause adverse immunologic response and conserve molecules favorable for tissue regeneration [68]. Onishi *et al*. [74] suggested that hMSC-derived growth factors, including bone morphogenetic protein(BMP)-2, vascular endothelial growth factor (VEGF) and basic fibroblast growth factor (bFGF), were preserved in decellularized ECM even after decellularization by freeze–thawing. In our study, though some of the signaling molecules were removed after the chemical decellularization and autoclaving, but remnant was still enough for bone regeneration. Besides, the Ca–P mineral components of the mECM further affected its stiffness and affinity to cytokines, which in turn will also boost osteogenesis [37, 39]. Overall, the higher osteogenesis in the mECM coated porous MCs is a result of bioactive molecules composed in ECM, porous structure and intrafibrillar Ca–P mineralization.

### Bone regeneration potential of mECM coated porous MCs *in vivo*

**Figure 8.**
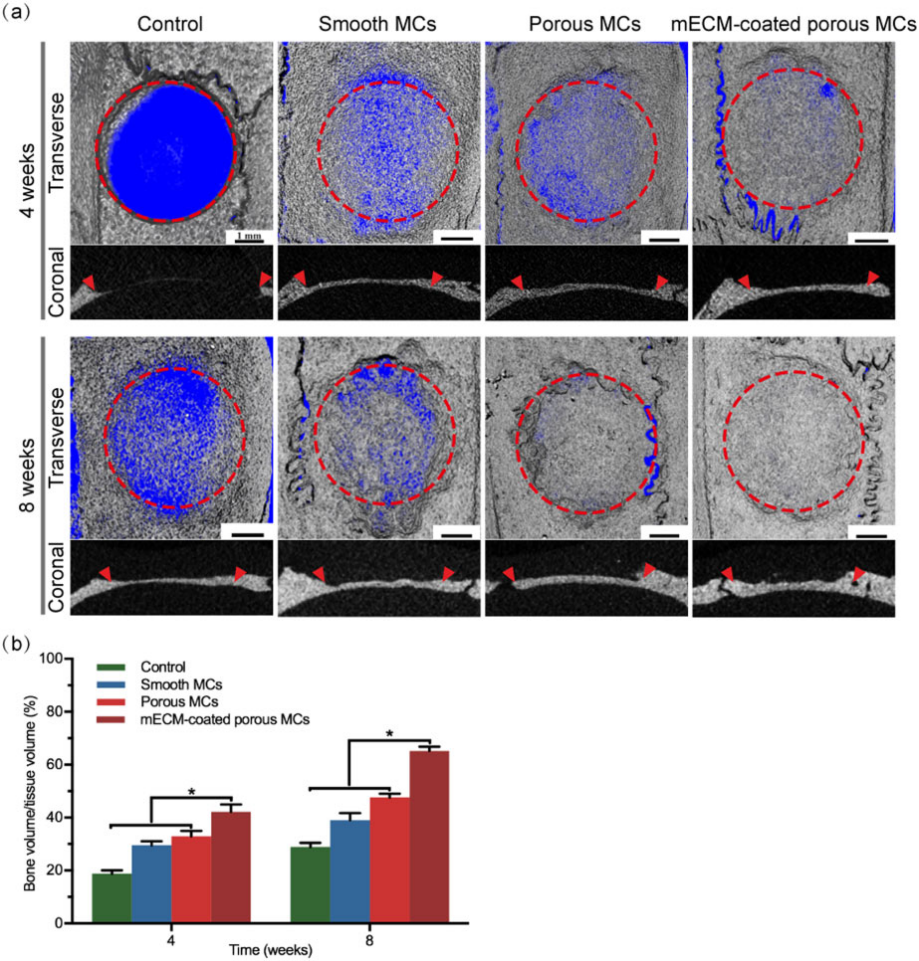
Bone regeneration in rat calvarial bone defects. (**a**) Representative micro-CT images of different samples post-transplantation in rat calvarial defects at 4 and 8 weeks, respectively. Red dashed circles and arrows indicated the space in which the original defect was created (all scale bar lengths are 1 mm). (**b**) Corresponding values of new BV/TV for various samples based on micro-CT data (*P ≤ 0.05, *n* = 3 for each group)

A rat calvarial bone defect model was used to evaluate the bone regeneration potential of the mECM coated porous MCs. Micro-CT scans were performed after 4 and 8 weeks of implantation. The representative images of different groups after reconstruction are shown in Fig. 8a. After 4 weeks of implantation, an obvious gap in bone growth is visible in the control, smooth and porous MCs; however, in the mECM coated MCs, the newly formed bone almost completely covered the defected area. After 8 weeks of implantation, the gap was still visible in both the control and smooth MCs group, while the new bone entirely covered the defect area both in the porous MCs and in mECM coated MCs groups. However, the mECM coated porous MCs group exhibited superior bone thickness and density.

Next, we evaluated the BV fraction (BV/TV) (Fig. 8b). After 4 and 8 weeks of implantation, the BV/TV values in the mECM coated MCs groups were 42.19 ± 2.80% and 65.19 ± 1.63%, respectively, which were significantly higher than the other groups.

In this study, cell-derived mECM coated porous MCs were fabricated to improve the bioactivity and osteogenesis of PEEK, which is regarded as bioinert material. They provided a physiological microenvironment conductive to cell adhesion, proliferation, ECM secretion and osteogenic differentiation. Additionally, they showed excellent bone regeneration outcomes in critical-sized defects without exogenous cells or growth factors. We suggest that these biomimetic designed highly interconnected porous mECM coated MCs have many promising applications in large-scale cell cultivation and injectable bone tissue engineering.

## Conclusions

A simple wet-chemistry strategy of hydroxylation was employed to bioactivate the PEEK MCs by allowing simultaneous modification of surface morphology and chemical composition. These modified MCs exhibited a uniform pore microstructure on the surface, which significantly improved cell adhesion, spreading, proliferation and ECM secretion compared to the smooth MCs. Moreover, mECM was successfully deposited on the surface of the porous MCs through repeated decellularization and recellularization strategy, which further promoted cell proliferation and osteogenic activity. Also, *in vivo* study in a rat model of bone defects revealed that mECM coated porous MCs performed better in bone regeneration compared to the other MCs. Overall, our results suggest that porous PEEK MCs with cell-derived mECM coatings have great potential in bone tissue engineering.

## Supplementary data

Supplementary data are available at *REGBIO* online.

*Conflict of interest statement.* All authors have declared that no support, financial or otherwise, has been received from any organization in this research.

## Supplementary Material

rbab013_Supplementary_Data
