# Supplementary material for: Porous polyetheretherketone microcarriers fabricated via hydroxylation together with cell-derived mineralized extracellular matrix coatings promote cell expansion and bone regeneration
Source: Regen Biomater. 2021 Mar 19;8(2):rbab013. doi: 10.1093/rb/rbab013 (PMC7975764; doi:10.1093/rb/rbab013)
Supplement: rbab013_Supplementary_Data [file rbab013_supplementary_data.zip › Supplementary data_revised.docx]

**Supplementary data**

**Porous polyetheretherketone microcarriers fabricated via**

**hydroxylation together with cell-derived mineralized extracellular matrix coatings promote cell expansion and bone regeneration**

Shuo Sun^1,2^, Zixue Jiao^2^, Yu Wang^2^, Zhenxu Wu^2^, Haowei Wang^1^, Qingming Ji^1,2^,

Yi Liu^1,^*, Zongliang Wang^2,^* and Peibiao Zhang^2,^*

^1^Department of Spine Surgery, The First Hospital of Jilin University, Changchun 130021, China; ^2^Key Laboratory of Polymer Ecomaterials, Changchun Institute of Applied Chemistry, Chinese Academy of Sciences, Changchun, 130022, China

*Corresponding author’s Email: [liuyi99@jlu.edu.cn](mailto:liuyi99@jlu.edu.cn) (Y.L.), [wangzl@ciac.ac.cn](mailto:wangzl@ciac.ac.cn) (Z.W.), [zhangpb@ciac.ac.cn](mailto:zhangpb@ciac.ac.cn) (P.Z.)

**Preparation of smooth PEEK MCs**

1.8 g PEEK powder was dissolved in 30 mL concentrated sulfuric acid by mechanical stirring and then the homogenous solution was dripped into 30% ethanol aqueous solution (1000 mL) as the substitutional solution at a constant nitrogen airflow rate (8 L/min) through a Teflon needle (27 G) to fabricate PEEK MCs with smooth surface. The MCs were immersed in deionized water for 48 h, and the water was changed every 8 h. Then the smooth PEEK MCs were hydrothermal treated with deionized water in the reactor (100 mL) at 170°C for 8 h. The smooth PEEK MCs were then vacuum dried and stored for further usage.

**Cytotoxicity test**

0.1 g PEEK MCs were immersed in 1 mL Dulbecco’s modiﬁed Eagle’s medium (DMEM) and transferred to a shaker at 150 rpm at 37°C for 24 h. The culture media extract was filtration sterilized by a syringe filter unit (Millex-GP, 0.22 μm, Millipore). MC3T3-E1 cells were seeded in 96-well plates with 8 $\times$ 10^3^ cells per well and cultured to adhere for 24 h. The culture media was then replaced with fresh culture medium (control), 100% (v/v) extract and 50% (v/v) extract (diluted with DMEM). After another 24 h incubation, 20 μL CCK-8 reagent was added to each well and continued incubating for 2 h at 37°C. Afterward, the absorbance intensity was measured by a microplate reader at 450 nm (Infinite M200, Tecan). The cytotoxicity of the samples was expressed as cell viability ratio, which was calculated by the following equation: Cell viability (%) = OD values (Samples)/OD values (Controls) × 100. The average value of three parallel samples was calculated as the final results.

**Cell proliferation**

MC3T3-E1 cells were cultured in 48-well plate at a density of 2 $\times$ 10^4^ for 1, 3 and 7 d, respectively. At designed time points, the medium was replaced with 500 μL fresh culture medium which contained 30 μL CCK-8. After incubation at 37°C for 2 h, 100 μL of incubated solution was then transferred to a 96-well plate and measured using a microplate reader at 450 nm (Infinite M200, Tecan).

**ALP staining and activity**

For ALP staining, after incubation of 7 days, the cell-seeded wells were fixed with 4% paraformaldehyde (PFA) at room temperature for 20 min. After washing with phosphate buffer solution (PBS) for 3 times, the cells were incubated with a mixture of BCIP/NBT solution at room temperature in the dark for 24 h according to the manufacturer’s protocol. After staining, the cell-seeded wells were washed twice with PBS and observed by stereo microscopy (MUOU^®^, China). For the ALP activity, the medium in each well was removed and rinsed once with PBS. Afterward, the cells were lysed using cell lysis buffer for Western and IP (Beyotime Biotechnology, Shanghai, China) with 1mM PMSF (phenylmethanesulfonyl fluoride, Solarbio^®^, Beijing, China) as protease inhibitor. After freezing and thawing twice, each of the lysates was centrifuged at 12000g at 4°C for 5 min to remove the cell debris. After centrifugation, 50 μL of the collected supernatant was used to react with 50 μL para-nitrophenyl phosphate (pNPP, Beyotime) at 37°C for 30 min in the dark. The reaction with pNPP was then quenched by addition of 100 μL terminating reagent. Absorbance at 405 nm was measured on a multifunctional microplate reader to quantify p-nitrophenol. BCA protein assay kit (Thermo Fisher Scientific, USA) was used to measure the total protein quantity at 562 nm for normalization. The corresponding ALP quantitative evaluation was calculated according to the ration of OD_405_/OD_562_.

**Alizarin red staining and calcium deposition assay**

For the alizarin red staining and calcium deposition assay, the cell-seeded microcarriers were rinsed with PBS twice, fixed with 4% paraformaldehyde for 20 min and then rinsed with PBS again. Afterward, 200 μL alizarin red solution was added to each well and incubated at 37°C for 2 min. After washed with PBS for several times, the cell-seeded wells were observed by stereo microscopy. For quantitative analysis, 10% cetylpyridinium chloride (CPC, 500 μL added to each well) was used to desorb calcium ions. After incubation at 37°C for 1 h, absorbance at 540 nm was read by a multifunctional microplate scanner.

**Quantitative real-time polymerase chain reaction (qRT-PCR)**

After incubation of 7 days, the cell-seeded wells were washed with PBS once and total RNA was extracted by Monzol Reagent (Monad) according to the protocol. The concentration and purity of RNA were measured using Nanodrop Plates (Tecan Infinite M200) and the RNA was reverse transcribed using PrimeScript RT Reagent Kit (Takara Biomedical Technology Co., Ltd). qRT-PCR was carried out by Stratgene Mx3005P (Agilent Technologies Inc., USA) and osteogenesis-related genes including runt-related transcription factor 2 (Runx2) and collagen type I (Col-I) were assessed. Primers were designed using Primer 5.0 software and commercially synthesized (Table S1). Glyceradehyde-3-phosphate dehydrogenase (GAPDH) was used as the reference gene for the evaluation of expression of target genes. The standard $\Delta\Delta$Ct (threshold cycles) method was used for the calculation of relative transcript quantities.

Table S1. Primer sequences for quantitative real-time polymerase chain reaction (qRT-PCR) of GAPDH, Runx 2 and Col-I

| Gene | Forward primer sequence (5'-3') | Reverse primer sequence (3'-5') |
| --- | --- | --- |
| GAPDH | ATGGTGAAGGTCGGTGTG | TGTAGTTGAGGTCAATGAAGGG |
| Runx2 | GCCGGGAATGATGAGAACTA | GGACCGTCCACTGTCACTTT |
| Col-I | CGCTGGCAAGAATGGCGATC | ATGCCTCTGTCACCTTGTTCG |

**
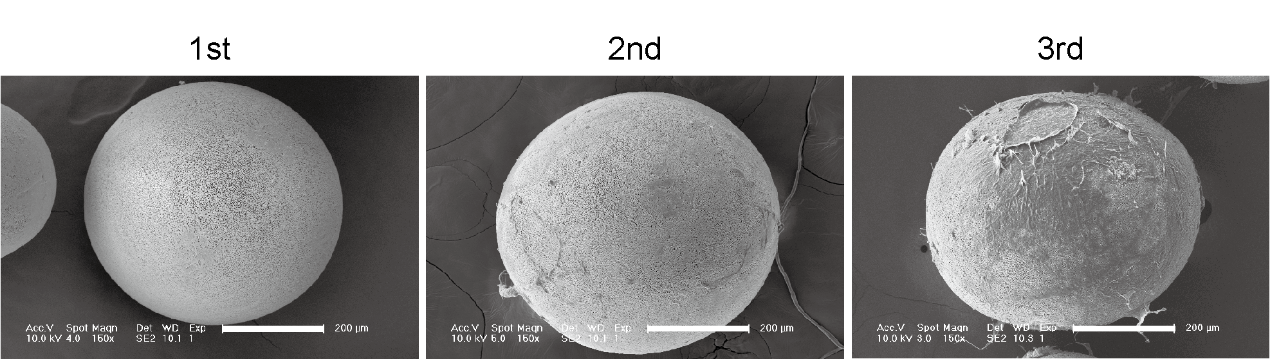
**

Figure S1. SEM images of porous microcarriers after different rounds of decellularization (All scale bar lengths are 200$\mu$m).

**
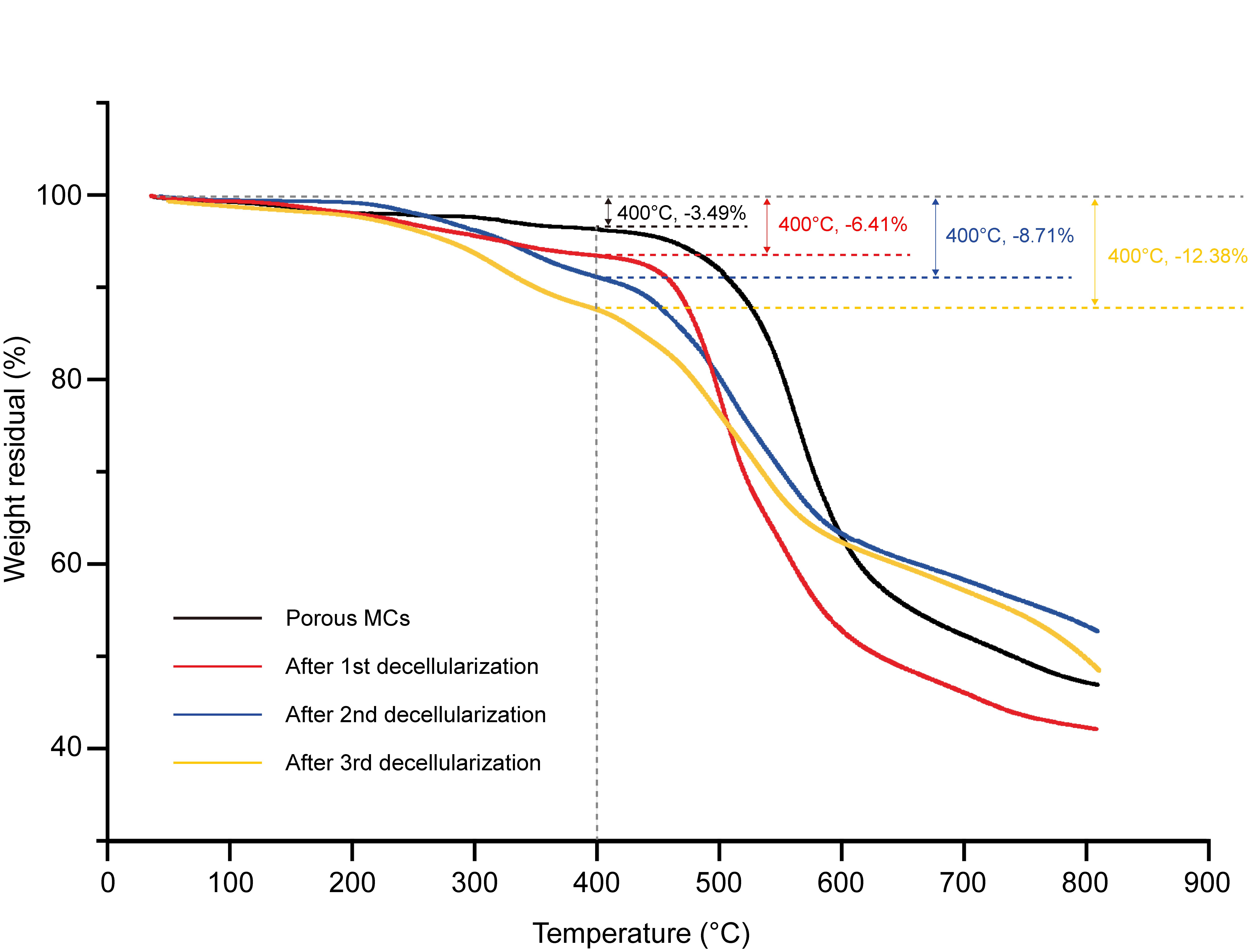
**

Figure S2. TGA (thermogravimetric analysis, TA Instruments TGA500, USA) curves of porous microcarriers after different rounds of decellularization. Samples of approximate 10mg were heated at the heating rate of 10$^{\circ}$C/min from 40$^{\circ}$C to 800$^{\circ}$C under nitrogen atmosphere.
