# Supplementary material for: Porous polyetheretherketone microcarriers fabricated via hydroxylation together with cell-derived mineralized extracellular matrix coatings promote cell expansion and bone regeneration
Source: Regen Biomater. 2021 Mar 19;8(2):rbab013. doi: 10.1093/rb/rbab013 (PMC7975764; doi:10.1093/rb/rbab013)
Supplement: rbab013_Supplementary_Data [file rbab013_supplementary_data.zip › Response to reviewers.docx]

**NOTE:** The revised contents in the manuscript were labeled in RED. Our responses were written in BLUE just after each comment (BLACK) of the reviewers. The revised content of manuscript was briefly shown following our response.

First of all, we would like to express our great appreciation to the reviewers for their helpful comments and suggestions.

**Response to reviewers’ comments**

**Reviewer: 1**

Comments:

The manuscript by Zhang et al. reported a mineralized extracellular matrix-coated porous PEEK microcarriers by selective wetchemistry strategy and recellularization/decellularization, and investigated their applications for bone regeneration. The properties of this microcarrier, including protein absorption, cytotoxicity, and acceleration on cell proliferation and osteogenic activity, were investigated. Finally, the properties for bone regeneration were verified by a rat calvarial defect model. This study introduced a novel strategy for the modification of PEEK porous microcarriers and presented some interesting results for bone regeneration application. However, there are still some concerns to be addressed before further consideration.

1. Introduction part, the authors should introduce the recent applications of PEEK for

the healing of calvarial defect, and analyze the critical points to determine its healing property.

**Response:**

We appreciate it very much for this good suggestion, and we have added the recent studies and discussed the critical properties of PEEK for the healing of calvarial defects in the introduction part. Some critical points of PEEK to determine its healing property, such as surface chemistry and morphology have been stated in paragraph 4 in the Introduction part.

“PEEK has been used more recently in calvarial reconstruction. Its advantage lies in minimal imaging artifact, being nonmagnetic, lightweight, and an inert nonconductor …”

“For example, the surface chemistry altered by functional groups and nano-/micro-patterned surfaces greatly influences cell adhesion, migration and differentiation…”

2. Were the surface hydrophilicities of PEEK smooth microcarriers and porous microcarriers investigated and compared?

**Response:**

We agree that more studies should be done to investigate the surface hydrophilicity of smooth and porous microcarriers. The static water contact angle measurement was performed to evaluate the surface hydrophilicity of different samples. As shown in Fig. 4c, the water contact angle was 91.08 $\pm$ 3.88$^{\circ}$ and 65.88 $\pm$ 4.55$^{\circ}$ for the smooth and porous microcarriers, respectively. On the basis of the results, it could be concluded that the porous PEEK microcarriers fabricated via hydroxylation improved surface hydrophilic property significantly.

“The static water contact angles of the smooth and porous MCs were measured by the sessile drop method on a contact angle system …”

“The static water contact angle measurement was used to compare the hydrophilicity of smooth and porous MCs (Fig. 4c). The water contact angle was 91.08 $\pm$ 3.88$^{\circ}$ for the smooth MCs, whereas it decreased to 65.88$\pm$ 4.55$^{\circ}$ after hydroxylation …”

**Figure 4c.** Static water contact angle of the smooth and porous microcarriers.

3. For the groups with different times of recellularization, the amounts of ECM on porous PEEK microcarriers should be different? Did the authors measure?

**Response:**

It is really a great suggestion as the reviewer pointed out that the amount of ECM on the porous PEEK microcarriers with different cycles of recellularization should be measured. As shown in the SEM images of Fig. 6, the ECM was better widespread and denser with increasing decellularization times, but this might not be accurate. Thermogravimetric analysis (TGA) was used to quantitatively measure the ECM amount and the results are shown in **Supplementary** Fig. S2. By dry weight, ECM is mainly composed of collagen[1] and most of the mass loss occurred below 400$^{\circ}$C from the TGA-curves of collagen samples[2]. Besides, medical grade PEEK-based materials exhibit melting temperature of around 340$^{\circ}$C; however, PEEK polymers demonstrate good melt stability at 400$^{\circ}$C and there was no thermal decomposition during the 3D printing process at this temperature[3]. Taken together, we regard the mass loss before 400$^{\circ}$C as the amount of ECM on the microcarriers in **Supplementary** Fig. S2. The mass loss of the pure porous microcarriers group was only 3.49% at 400$^{\circ}$C. With the increase of decellularization cycles, the mass loss was changed from 6.41% to 12.38%.

“Thermogravimetric analysis (TGA, TA Instruments TGA500, USA) was also used to quantitatively measure the ECM amount and the results are shown in **Supplementary** Fig. S2.”

Figure S2. TGA (thermogravimetric analysis, TA Instruments TGA500, USA) curves of porous microcarriers after different rounds of decellularization. Samples of approximate 10mg were heated at the heating rate of 10$^{\circ}$C/min from 40$^{\circ}$C to 800$^{\circ}$C under nitrogen atmosphere.

**Reviewer: 2**

General comments:

It is interesting paper.

In this study, porous PEEK microcarriers coated with mineralized extracellular matrix were developed for orthopedic applications. Porous PEEK microcarriers were derived from smooth microcarriers using a simple selective wet-chemistry strategy involving the reduction of carbonyl groups. The deposition of mECM was achieved by repeated cellularization and decellularization on the surface of porous microcarriers. The obtained mECM coated microcarriers exhibited excellent bone regeneration capacity in a calvarial defect rat model in vivo.

Special comments:

1. Please provide the SEM images of the microcarriers at different cellularization-decellularization cycle for comparison.

**Response:**

It is really a good idea as the reviewer suggested, and we have provided SEM images of the porous PEEK microcarriers after different cycles of cellularization-decellularization in **Supplementary** Fig. S1. Actually, as low magnification SEM images shown in Fig. 6, the extracellular matrix on the surface of microcarriers was better widespread and denser with increasing decellularization times, but just as the reviewer pointed out, the panorama SEM images of microcarriers at different cellularization-decellularization cycle may be better for comparison, so we added this part in the **Supplementary data**.

“We noticed that mECM was better widespread and denser with increasing decellularization times (also in **Supplementary** Fig. S1).”

Figure S1. SEM images of porous microcarriers after different rounds of decellularization (All scale bar lengths are 200$\mu$m).

2. Figure 6(a), the rightmost panel: “Calccein AM” should be corrected to “Calcein AM”

**Response:**

We are very sorry for our incorrect writing and it is rectified in Fig. 6a.

Figure 6. Characterization and recellularization evaluation of the mECM coated porous microcarriers …

Additionally, some words, punctuation marks, and references were corrected. All of these revisions were labeled by the ‘track change’ mode in the revised manuscript. We thank all the reviewers again and remain at your disposal for any further questions.

References

1.	Badylak SF, Freytes DO, Gilbert TW. Extracellular matrix as a biological scaffold material: Structure and function. Acta Biomater 2009;5:1-13.

2.	Lozano LF, Pena-Rico MA, Heredia A et al. Thermal analysis study of human bone. Journal of Materials Science 2003;38:4777-4782.

3. Zanjanijam AR, Major I, Lyons JG et al. Fused Filament Fabrication of PEEK: A Review of Process-Structure-Property Relationships. Polymers 2020;12.
